# Going Green, but Staying in the Black: How Framing Impacts the Agreement With Messages on the Economic Consequences of Environmental Policies

**DOI:** 10.3389/fpsyg.2021.624001

**Published:** 2021-04-12

**Authors:** Mauro Bertolotti, Patrizia Catellani

**Affiliations:** Department of Psychology, Catholic University of Milan, Milan, Italy

**Keywords:** message framing, pro-environmental attitudes, policy evaluation, place identity, economic impact, trade off between economy and ecology

## Abstract

Past research suggests that although citizens are generally favorable to pro-environmental policies, their negative economic impact can be a relevant source of concern. In two studies, we investigated the agreement with messages highlighting the positive vs. negative economic impact of a pro-environmental policy (the creation of a protected natural reserve in a lakeside area), as a function of the framing of the policy itself in terms of local relevance (Study 1) and environmental impact (Study 2). In Study 1, participants (*N* = 514) were citizens of different Italian regions. Results showed that reference to the local (vs. global) relevance of the proposed policy increased the tendency to agree with loss-framed (vs. gain-framed) messages on the economic impact of the policy. In Study 2, participants (*N* = 500) were a sample of actual lakeside residents from the Garda Lake area in Italy. Results showed that reading messages promoting the policy through stressing the negative consequences of not implementing it (vs. the positive consequences of implementing it) increased the tendency to agree with a subsequent loss-framed (vs. gain-framed) message on the economic impact of the policy. This effect was more evident among participants with stronger place identity. Discussion focuses on the relevance of framing and matching effects in devising persuasive messages on the environmental and economic impact of pro-environmental policies.

## Introduction

Despite the increasing centrality of environmental issues in the public debate (Lorenzoni and Pidgeon, 2006; Gifford, 2011; Pidgeon, 2012), the complexity and technicality of the matter often prevent citizens from forming accurate opinions, leaving them with superficial and ambivalent attitudes toward pro-environmental policies. On the one hand, most people agree on their urgency and the environmental benefits deriving from them. On the other hand, when the cost of the policies and their potential negative impact on economic development are discussed, many are reluctant to endorse them. This ambivalence can be sometimes exploited by politicians and interest groups, who may insist on the financial burden of pro-environmental policies in order to delay their adoption or reduce their scope (Boykoff, 2013).

In this paper, we tested how the framing of messages on the economic impact of pro-environmental policies can affect citizens’ evaluation of these policies. In two studies, we investigated the framing effects of messages describing the economic impact of the creation of a protected natural reserve in a lakeshore area. Study 1 was carried out with a nation-wide sample of Italian citizens. Study 2 was carried out with a sample of inhabitants from the area of Garda Lake, the largest Italian lake. Our aim was to test to what extent, and under what conditions, participants exposed to a message anticipating a positive vs. negative economic impact of a pro-environmental policy (i.e., gain vs. loss framing) would agree with the message.

In Study 1, we expected that reading a message including an explicit reference to the *local* (*vs*. *global*) relevance of the proposed environmental policy would increase the tendency to agree with the same message framing the expected economic impact of the policy as a *loss* (*vs*. *gain*). In Study 2, we expected that reading a message stressing the *negative* environmental consequences of *not implementing* the proposed policy, i.e., another form of *loss* framing (vs. the *positive* consequences of *implementing* it, i.e., *gain* framing) would increase the tendency to agree with a subsequently presented message again framing the expected economic impact of the policy as a *loss* (*vs*. *gain*). We also expected that this tendency would be more evident among participants with stronger place identity. A confirmation of these results would contribute to increase our knowledge of which framing and individual factors heighten citizens’ perception of the *negative* economic impact of pro-environmental policies.

### Framing the Impact of Pro-environmental Policies

Several studies indicate that citizens are increasingly aware of environmental risks, such as pollution, climate change, and excessive land use (Lorenzoni and Pidgeon, 2006; Lee et al., 2015). Given the perceived relevance of such risks, most citizens are also generally favourable toward public action aimed at dealing with them. By selecting and organizing the information they provide to citizens regarding the motivations, and, crucially, the impact of pro-environmental policies, policymakers, and the media *frame*, these policies (Gamson and Modigliani, 1989; Entman, 1993; Scheufele, 1999) attributing them different meanings and interpretations. The most frequently form of framing studied so far is *valence framing*, which consists in framing a proposed policy or behavior by highlighting its positive (*gain frame*) or negative (*loss frame*) impact.

Some research has showed that loss-framed messages tend to be more persuasive than gain-framed messages (Kahneman and Tversky, 1979; Tversky and Kahneman, 1981; Meyerowitz and Chaiken, 1987), as individuals are generally risk-averse, and thus motivated to avoid negative outcomes more than they are motivated to seek positive outcomes (Tannenbaum et al., 2015). This tendency has been observed also in the case of environmental issues (Davis, 1995; Nisbet, 2009; Scrase and Ockwell, 2010; Van de Velde et al., 2010). Consistently, pro-environmental policies are often presented in the media with a loss-framed approach (Hulme, 2008; Nisbet, 2009; Cox, 2010; McDonald, 2013). Environmental action is primarily framed as means to avoid negative environmental impact, such as pollution, biodiversity loss, or global warming and climate change (Reber and Berger, 2005; Moser and Dilling, 2007). This type of framing has the advantage of simultaneously raising awareness of the relevance and scope of environmental problems, which not all citizens may know about, and providing a potential solution to them, in the form of the proposed policies.

Recent research on message framing in environmental communication (Bertolotti and Catellani, 2014, 2015) and in other areas (Cesario et al., 2013) has suggested that multiple levels of framing can be used simultaneously. These different levels of framing are associated with different individual self-regulatory orientations, resulting in different effects on individuals’ judgments and decisions. It is therefore important to look at how pro-environmental policies are discussed, as multiple competing frames can tinge the public debate.

### The Economic Impact of Pro-environmental Policies

As mentioned above, communication on pro-environmental policies can focus on varying dimensions of the policy, and one of them is the economic dimension. While gain-framed messages will focus on the economic benefits that may derive from implementing a pro-environmental policy, loss-framed messages will focus on the costs of adopting such policy (DeGolia et al., 2019). Pro-environmental policies can indeed have relevant economic costs, in the form of increased public spending (e.g., for infrastructure building, maintenance, or conversion), new taxes (e.g., carbon-taxes), or reduced profits for certain economic sectors (e.g., due to governments disincentivizing, regulating, or restricting certain uses of land, water, and air).

Under certain circumstances, such as an economic recession, the costs of pro-environmental policies may hinder local administrations’ capacity to adopt them (Kahn and Kotchen, 2011; Bechtel and Scheve, 2013). The awareness of these costs may also hinder popular support for pro-environmental policies. Although citizens may be willing to avoid negative environmental impacts of climate change, pollution, and excessive land use, they may also be concerned by the prospect of an economic loss resulting from the policies that address those issues. There is robust evidence of a positive correlation between economic wealth (or lack thereof) and public support for environmental policies (Franzen and Meyer, 2010). Data from international surveys such as the European Social Survey and the World Value Survey show that people tend to prioritise environmental issues only after a certain level of wealth is achieved (Franzen, 2003; Baek, 2015; Pisano and Lubell, 2016). Transient economic downturns and financial difficulties also result in reduced concern for environmental issues (Guber, 2003; Brulle et al., 2012; Scruggs and Benegal, 2012; Carmichael and Brulle, 2016). This is consistent with hierarchy of needs theory of Maslow (1954), according to which the need for economic security occupies a basic level in the needs pyramid. Political leaders opposing pro-environmental policies can highlight this potential trade-off by putting environmental and economic concerns against each other (Jacques et al., 2008). The resulting ambivalence might be resolved by certain communicational strategies that may “tip the balance” in citizens’ decision-making, making them perceive that the environmental benefits of pro-environmental policies outweigh their economic costs.

### The Role of Perceived Geographic, Temporal, and Social Distance

The perceived distance of the economic and environmental concerns may play an important role in how citizens evaluate the different arguments that are presented and discussed in the political debate on pro-environmental policies. Past research has suggested that framing climate change and other environmental issues as local (rather than global) problems can increase citizens’ involvement in pro-environmental behaviours and policies (e.g., Leiserowitz, 2007; O’Neill and Hulme, 2009; but see also Uzzell, 2000 for an opposite effect, termed “environmental hyperopia”). This is consistent with the idea that psychological distance from environmental risks, due to their perceived complexity, remoteness in time and place, and uncertainty, can hinder citizens’ concern (McClure et al., 2007; Viscusi et al., 2008), as well as their support for policies and individual behaviours addressing them. Subsequent research, however, has showed that simply highlighting the local consequences of environmental issues may not always work as intended (Spence and Pidgeon, 2010; Brügger et al., 2015).

Besides physical and temporal closeness, individuals’ cognitive and emotive attachment with their local natural and social environment is a key factor in citizens’ motivation to engage in pro-environmental action (Bonaiuto et al., 1996; García-Mira et al., 2005; Devine-Wright, 2009; Scannell and Gifford, 2013). Place identity, in particular, refers to the “physical world socialization of the self” (Proshansky et al., 1983, p. 57) and it has been conceptualised as one of the components of individuals’ identity, together with social identity (Jorgensen and Stedman, 2001; Twigger-Ross et al., 2003). Research in environmental psychology has shown that local place identity influences people’s perceptions of the area they live in, nature conservation behaviour (Giuliani and Scopelliti, 2009), as well as participation in collective action aimed at defending or restoring the natural environment in their area (Lemée et al., 2019). Some studies, however, have also suggested the potential downsides of a strong place identity, such as its negative association with the perception of environmental degradation in one’s local area, which can in turn decrease the intention to address the causes of such degradation (Walker et al., 2015).

When an economic loss is prospected in addition or as an alternative to an environmental risk, the activation of a local frame and citizens’ own identification with their place of residence may have unintended consequences. Locally-framed communication on environmental issues may raise attention and awareness of the problem. At the same time, however, when evaluating a policy with potential negative economic effects on a local scale, individuals may become more cautious than when evaluating a similar policy on a wider (e.g., national or global) scale. And individuals with a strong place identity, and thus greater attachment to their local community, may be even more reluctant to endorse a policy with negative economic repercussions on that same community (Devine-Wright, 2009; Read et al., 2013).

### Research Overview and Hypotheses

In the present research, we explored whether some dimensions of message framing can influence the agreement with messages on the economic impact of a pro-environmental policy. As the object of our analysis, we chose a land use regulation policy, i.e., the creation of a fictional natural reserve in a lakeside area. This type of policy is a typical case in which environmental and economic concerns can be at odds with each other (De Groot et al., 2010; Guerry et al., 2015), making the framing of the economic impact especially relevant. We analysed whether exposure to messages anticipating a positive (gain frame) vs. negative (loss frame) economic impact of the policy implementation would affect support for the policy.

In Study 1, we provided participants of a nation-wide survey with gain- vs. loss-framed messages on the expected economic impact of the institution of a natural reserve in a lakeside area. In addition to the gain vs. loss framing, we manipulated the local vs. global framing of the message, presenting the expected economic outcomes as relevant for the local vs. global community. We then measured participants’ agreement with the proposed policy, as well as their agreement with the message on the economic consequences of it. Starting from the abovementioned research results regarding the tendency to attach greater importance to the economic dimension of an environmental policy when it touches people’s everyday life closely (Read et al., 2013; Kachi et al., 2015; Mildenberger and Leiserowitz, 2017), we formulated the following hypothesis.

*H1*: Participants agree with loss-framed messages on the economic impact of a pro-environmental policy more when the impact refers to a local dimension than when it refers to a global dimension (while this is not the case for gain-framed messages).

In Study 1, we also assessed individual features of citizens known from previous research to affect support for pro-environmental policies, such as environmental risk perception and political orientation. Consistent with previous research, we expected that these factors would be associated with support for the policy, with participants perceiving more risk and left-wing oriented participants being more inclined to support the pro-environmental policy. However, we did not expect that risk perception and political orientation would significantly moderate the effect described in H1.

In Study 2, we further investigated the effects of a gain- vs. loss-framed message on the economic impact of a pro-environmental policy. Participants were a representative sample of residents in a lakeside area. In this case, before presenting the gain- vs. loss-framed message on the economic impact of the policy, we asked participants to read a message *in favour* of the introduction of the pro-environmental policy, *but* framed in two different ways: (a) emphasising the potential *benefits* to the lakeside area in case the policy was implemented (*gain* framing); (b) emphasising the potential *damages* to the lakeside area in case the policy was *not* implemented (*loss* framing). As in Study 1, we then measured participants’ agreement with the gain- vs. loss-framed message on the economic impact of the policy. We expected that being exposed to the loss-framed message regarding the environmental policy would increase the perceived fit with the loss-framed message on the economic impact of the policy. This would be consistent with past research on message framing (Cesario et al., 2013) and the framing of pro-environmental policies in particular (Bertolotti and Catellani, 2014). We therefore formulated the following hypothesis.

*H2*: Participants agree with loss-framed messages on the economic impact of a pro-environmental policy more after exposure to a loss-framed message supporting the policy rather than a gain-framed message supporting the policy. This is not the case for agreement with gain-framed messages.

In Study 2, we also assessed participants’ place identity. As discussed above, place identity may heighten citizens’ attention to potential threats to their local environment and community (Devine-Wright, 2009). Consistently, we expected the prospect of an environmental-economic trade-off to be especially concerning for citizens who feel strong ties with their place and community. These citizens would be particularly sensitive when both environmental and economic dangers are evoked. We therefore formulated the following hypothesis.

*H3*: The moderating effect of environmental loss framing on the persuasiveness of economic loss-framed messages (see H2 above) would be further increased among participants with strong (vs. weak) place identity.

## Study 1

### Participants and Procedure

Participants were a subset of the ITANES 2018 electoral survey panel.^1^ They completed an online questionnaire containing several questions on their voting intentions, political attitudes, and opinions. In addition, they were randomly allocated to some survey experiments. Participants allocated to the survey experiment presented in this paper were 514 (57.6% were women, ranging between 19 and 90 years old, *M* = 46.1, *SD* = 13.6).

In the experiment, participants were asked to imagine they lived near a lake and were presented a short description (73 words) of a policy proposal regarding the creation of a natural reserve in the lakeside area. They were then asked to evaluate said policy. After that, participants were presented with a fictitious politician’s response to the proposal, focusing on the expected economic impact of the policy implementation. In the text, the economic impact was presented as either positive or negative (*gain/loss frame*) and as focused on either a local or a more global reality (*local/global frame*). The resulting four different versions of the politician’s response are reported in Table 1. Participants were randomly assigned to read one of the four versions. Participants’ distribution across experimental conditions was as follows: gain local frame condition (*N* = 115), loss local frame condition (*N* = 137), gain global frame condition (*N* = 113), and loss global frame condition (*N* = 149). After reading the politician’s response, participants were asked to rate their agreement with the message.

**Table 1:** Messages on the economic consequences of the policy, manipulated as a function of gain/loss frame and local/global frame.

		Local/global frame		
		Local	Global
Gain/loss frame	Gain	“I am in favour of this proposal. If we adopt this plan for the creation of a *protected area in our lake’s territory*, we will *get* economic *benefits*, we will *improve* the quality of agricultural and industrial production, and we will *create* new jobs *in the land around our lake*.”	“I am in favour of this proposal. If we adopt plans like this for the creation of *protected areas*, we will *get* economic *benefits*, we will *improve the quality* of agricultural and industrial production, and we will *create* new jobs *everywhere*.”
	Loss	“I am against this proposal. If we adopt this plan for the creation of a *protected area in our lake’s territory*, we will *suffer* economic *damages*, we will *stop* agricultural and industrial production, and we will *lose* jobs *in the land around our lake*.”	“I am against this proposal. If we adopt plans like this for the creation of *protected areas*, we will *suffer* economic *damages*, we will *stop* agricultural and industrial production, and we will *lose* jobs *everywhere*.”

This order of presentation of the messages aimed at recreating a public debate format, with an initial proposal (the policy description) and subsequent arguments in support or against the proposal (the gain- vs. loss-framed messages on the economic impact).

### Measures

#### Agreement With the Pro-environmental Policy Proposal

Participants rated their agreement with the policy proposal and its expected positive impact using a 10-point scale ranging from 1 (“Completely disagree”) to 10 (“Completely agree”).

#### Agreement With the Gain- vs. Loss-Framed Message on the Economic Impact of the Policy

Participants rated their agreement with the message on the economic impact of the policy on a 10-point scale ranging from 1 (“Completely disagree”) to 10 (“Completely agree”).

#### Environmental Risk Perception

A single item measured participants’ perception of excessive land use as an environmental risk. The statement was as follows: “You may have heard that the increasing occupation of land for residential, commercial, industrial buildings, and infrastructures threatens the ecological equilibrium of the territory. To what extent do you think that excessive land use is a threat?” Response options ranged from 1 (“Completely disagree”) to 10 (“Completely agree”).

#### Political Orientation

Participants were asked to indicate their political orientation using an 11-point scale ranging from Left (1) to Right (11). An additional “none of the above” option was included.

#### Socio-Demographic Variables

Participants answered to questions regarding their gender, age, and education level (recoded as the number of years of school attendance from primary to tertiary education).

### Results

#### Predictors of Agreement With the Pro-environmental Policy Proposal

We first ran a hierarchical linear regression with agreement with the policy proposal as the dependent variable, and socio-demographic variables, political orientation, and environmental risk perception as predictors (Table 2, left side). No effect of gender, age, education, or political orientation emerged, while environmental risk perception was strongly and positively associated with participants’ agreement with the proposal, *β* = 0.396, *t* = 7.69, *p* < 0.001.

**Table 2:** Predictors of participants’ agreement with the policy proposal (Studies 1 and 2).

	Study 1			Study 2		
	*β*	*t*	*p*	*β*	*t*	*p*
Gender	0.071	1.40	0.161	−0.052	−1.33	0.185
Age	0.063	1.20	0.232	−0.038	−0.91	0.361
Education	0.047	0.87	0.384	−0.138	−3.33	0.001
Political orientation	−0.054	−1.02	0.310	0.061	1.56	0.120
Environmental risk perception	0.396	7.69	0.000	0.500	12.68	0.000
*R*^2^	*0.168*			*0.264*		
Place identity				0.326	7.32	0.000
*R*^2^				*0.335*		

#### Predictors of Agreement With the Message on the Economic Impact of the Policy

To test our main hypothesis, we investigated the predictors of participants’ agreement with the message on the economic impact of the policy proposal. We ran a hierarchical linear regression with agreement as the dependent variable, and three groups of predictors: gender, age, education, political orientation, and risk perception (Step 1); the manipulated variables, namely, economic impact frame (contrast-coded +1 for the gain frame condition and −1 for the loss frame condition) and local/global frame (contrast-coded +1 for the local frame condition and −1 for the global frame condition; Step 2); the interaction between the gain/loss and the local/global frames (Step 3).

**Figure 1:**
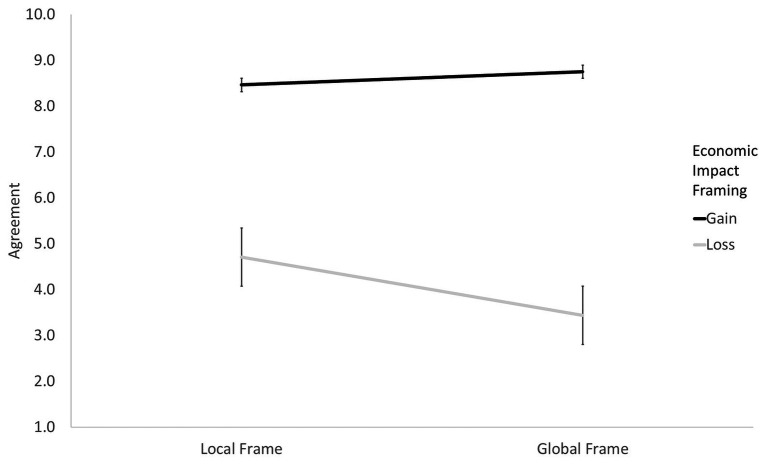
Agreement with gain vs. loss messages on the economic impact of the policy as a function of local vs. global framing (Study 1).

The results of the analysis are reported in Table 3. In Step 1, no effects of socio-demographic variables, political orientation, and environmental risk perception emerged. In Step 2, a large effect of the economic impact frame, *β* = 0.683, *t* = 16.60, *p* < 0.001, and a smaller effect of the local/global frame, *β* = 0.082, *t* = 2.02, *p* = 0.045, emerged. Participants agreed with the politician’s message more when it described the positive rather than the negative economic impact of the policy. To a much lesser extent, they also agreed more with the politician’s message when it was focused on a local reality rather than a global one. In Step 3, a significant interaction effect between the gain/loss and the local/global frames emerged, *β* = −0.121, *t* = 3.00, *p* = 0.003, fully confirming our H1 (Figure 1). The message describing the negative economic impact of the pro-environmental proposal (loss-framed message) was significantly more effective when this impact was embedded in a local frame than when it was embedded in a global frame (*M* = 4.71, *SD* = 3.00 and *M* = 3.44, *SD* = 2.41, respectively), *p* < 0.001. When the message described the positive economic impact of the proposal (gain-framed message), the difference between the text embedded in a local frame and the text embedded in a global frame was instead not significant (*M* = 8.47, *SD* = 2.18 and *M* = 8.75, *SD* = 1.96, respectively), *p* = 0.410.

**Table 3:** Predictors of participants’ agreement with the statement on the economic impact of the policy (Study 1).

		*β*	*SE*	*β*	*t*	*p*
*Step 1*	(Constant)	7.065	1.439		4.910	0.000
	Gender	0.330	0.379	0.049	0.869	0.385
	Age	−0.017	0.014	−0.071	−1.233	0.219
	Education	−0.030	0.056	−0.031	−0.524	0.600
	Political orientation	−0.050	0.059	−0.049	−0.841	0.401
	Envir. risk perception	0.095	0.191	0.028	0.496	0.620
*Step 2*	(Constant)	6.134	1.058		5.795	0.000
	Gender	−0.185	0.281	−0.027	−0.660	0.510
	Age	−0.001	0.010	−0.003	−0.074	0.941
	Education	0.032	0.042	0.034	0.781	0.435
	Political orientation	0.014	0.043	0.014	0.317	0.752
	Envir. risk perception	0.025	0.140	0.008	0.181	0.857
	Gain/loss framing	2.326	0.140	0.683	16.593	0.000
	Local/global framing	0.277	0.137	0.082	2.015	0.045
*Step 3*	(Constant)	5.963	1.047		5.695	0.000
	Gender	−0.143	0.278	−0.021	−0.514	0.608
	Age	0.000	0.010	0.001	0.021	0.983
	Education	0.035	0.041	0.037	0.863	0.389
	Political orientation	0.019	0.043	0.019	0.443	0.658
	Envir. risk perception	0.007	0.139	0.002	0.054	0.957
	Gain/loss framing	2.320	0.138	0.681	16.756	0.000
	Local/global framing	0.231	0.137	0.068	1.693	0.092
	Gain/loss × Local/global framing	−0.410	0.137	−0.121	−3.003	0.003

Gain/loss framing coded as +1 gain, −1 loss; Local/global framing coded as +1 local, −1 global.

To check whether the above interaction effect was moderated by other factors, we carried out a further analysis (Step 4) introducing all three-way interactions among economic impact frame, local/global frame, political orientation, and environmental risk perception. No significant effects were found, *βs* < 0.033, *t*s < 0.95, *p*s > 0.345.

In sum, participants’ perception of a severe environmental risk (in this case, excessive land use) was the main factor predicting their agreement with the pro-environmental policy proposal, whereas participants’ agreement with the message on the economic impact of the policy varied as a function of the local/global and gain/loss framing. Participants generally agreed more with the message describing the potential economic gains than with the message describing the potential economic losses. However, this gap was significantly reduced when the negative economic impact was embedded in a local frame than when it was embedded in a global frame. This result suggests that communication aimed at contrasting the adoption of pro-environmental policies by stressing their negative economic impact may indirectly benefit from citizens’ concern for the welfare of their local community. In Study 2, we further explored this finding, investigating the role of the framing of the environmental consequences of policies and citizens’ identification with their local environment.

## Study 2

### Participants and Procedure

Participants were 500 Italian residents of small- and medium-sized communities near Lake Garda, who were contacted as part of the CLIC-Plan research project.^2^ Their age ranged between 18 and 87 years old (*M* = 39.7, *SD* = 14.7), with balanced gender representation (53.6% females, 46.4% males). Participants were asked to complete an online questionnaire containing a survey experiment, as well as several questions on their pro-environmental and political attitudes.

In the experiment, participants were asked to imagine that the administration of the town in which they lived was proposing a plan to create a protected natural area of 30 ha on the shores of the lake. They read a short text (approximately 50-word long) *in favour* of the policy implementation, *but* framed in two different ways: (a) emphasising the potential *benefits* to the lakeside area in case the policy was implemented (*gain* framing); (b) emphasising the potential *damages* to the lakeside area in case the policy was *not* implemented (*loss* framing). Participants then read a reaction to the proposal (approximately 100-word long) attributed to a group of citizens, and focused on the economic impact of the creation of the natural reserve. This impact was presented in either a gain or a loss frame. The full text of the resulting four versions of the message is reported in Table 4. Participants were randomly assigned to read one of these versions namely the environmental loss and economic loss frame condition (*N* = 134), environmental loss and economic gain frame condition (*N* = 111), the environmental gain and economic loss frame condition (*N* = 121), and the environmental gain and economic gain frame condition (*N* = 134).

**Table 4:** Framing of the pro-environmental policy proposal and of the economic impact message.

Frame of the pro-environmental policy	
Gain	Loss
The current administration claims that if the municipality *adopts* these measures, there will be considerable environmental *benefits*. The reduction of land use around the lake will *protect and improve* the natural landscape, *improve* the quality of lake waters, and *preserve* the hydrogeological balance.	The current administration claims that if the municipality *does not adopt* these measures, there will be considerable environmental *damages*. The increase of land use around the lake will *alter and compromise* the natural landscape, *decrease* the quality of lake waters, and *threaten* the hydrogeological balance.
Frame of the economic impact message	
Gain	Loss
This proposal has sparked a debate among citizens of your municipality and many citizens are in favour of the proposal. They think that if a natural reserve is created, economic *benefits* will be obtained. In particular, if the area becomes protected, sustainable agricultural and industrial production will be *encouraged*. As a result, new jobs may be *created*. Furthermore, if a natural reserve is established, a more sustainable form of tourism will be *promoted*, with *positive* effects on economic growth. In conclusion, these citizens think that, if this proposal is adopted, it will bring *advantages* to tourism, employment, and the economy as a whole.	This proposal has sparked a debate among citizens of your municipality and many citizens are against the proposal. They think that if a natural reserve is created, economic *damages* will be caused. In particular, if the area becomes protected, current agricultural and industrial production will be *impaired*. As a result, jobs may be *lost*. Furthermore, if a natural reserve is established, mass tourism will be *limited, with negative* effects on economic growth. In conclusion, these citizens think that, if this proposal is adopted, it will bring *disadvantages* to tourism, employment, and the economy as a whole.

### Measures

#### Manipulation Check

Two questions at the end of the first section of the questionnaire were used to check participants’ understanding of the stimuli. The first question investigated the comprehension of the policy proposal framing, by asking participants to indicate whether the proposal to institute a natural reserve mentioned “the environmental *benefits* that could derive from its adoption” or “the environmental *damages* that could derive from not adopting it,” representing, respectively the environmental gain- or loss-frame conditions. The second question investigated the comprehension of the economic impact message framing, asking participants to indicate whether the text they read highlighted “the economic *benefits* deriving from the institution of a natural reserve” or “the economic *damages* deriving from the institution of a natural reserve,” representing the gain-frame and the loss-frame economic conditions, respectively.

#### Agreement With the Pro-environmental Policy Proposal

Participants rated their agreement with the initial pro-environmental policy proposal using a 10-point scale ranging from 1 (“Completely disagree”) to 10 (“Completely agree”).

#### Agreement With the Message on the Economic Impact of the Policy

Participants rated their agreement with the message on the economic impact of the implementation of the policy proposal on a seven-point scale, ranging from 1 (“Completely disagree”) to 7 (“Completely agree”).

#### Environmental Risk Perception

A single item measured participants’ perception of excessive land use as an environmental risk in their area. The statement was as follows: “You may have heard that the increasing occupation of land for residential, commercial, industrial buildings, and infrastructures threatens the ecological equilibrium of the territory. To what extent do you think that excessive land use is a threat?” Response options ranged from 1 (“Completely disagree”) to 7 (“Completely agree”).

#### Place Identity

Participants’ place identity was measured with eight items adapted from Scannell and Gifford (2010). Examples of the items are: “I feel to be part of the community of Lake Garda,” “This lake is special for me,” and “I am proud of my lake.” Participants’ agreement with the statements contained in the items was assessed using a seven-point scale ranging from 1 (“Completely disagree”) to 7 (“Completely agree”). A single place identity index was then computed, *α* = 0.957.

#### Political Orientation

Participants’ political orientation was measured with a single item asking them to indicate their position using an 11-point scale ranging from Left (1) to Right (11). An additional “none of the above” option was included.

#### Socio-Demographic Variables

We measured participants’ gender, age, and education level.

### Results

#### Manipulation Check

Most participants (95.3% in the environmental gain condition, 89.8% in the environmental loss condition) correctly identified the environmental impact framing of the first message, the one describing the policy, *χ*^2^ (1, *N* = 500) = 363.76, *p* < 0.001. This was also the case for the identification of the economic impact framing of the second message, the one describing the reactions to the policy (94.3% in the economic gain condition, 85.9% in the economic loss condition), *χ*^2^ (1, *N* = 500) = 325.72, *p* < 0.001.

#### Predictors of Agreement With the Pro-environmental Policy Proposal

We analysed the predictors of agreement with the pro-environmental policy proposal through a hierarchical linear regression (Table 2, right side), with socio-demographic variables, political orientation, and perceived environmental risk as predictors in Step 1, and place identity and the framing of the policy proposal (contrast-coded +1 for the gain condition and −1 for the loss condition) in Step 2.^3^ As in Study 1, no significant effects of gender, age, and political orientation emerged, while environmental risk perception was again strongly and positively associated with support for the policy, *β* = 0.500, *t* = 12.68, *p* < 0.001. In Step 1, an unexpected negative effect of education was also found, *β* = −0.138, *t* = 3.38, *p* = 0.001. In Step 2, no significant effect of the framing of the environmental impact of the policy emerged, while participants’ place identity turned out to have a significant and positive effect on agreement with the proposal, *β* = 0.325, *t* = 7.31, *p* < 0.001. Thus, results showed that agreement with the pro-environmental policy was driven by participants’ perception of the environmental risk, but also by place identity, indicating that participants who felt closer to the place they lived in were more willing to protect it by supporting the policy. The gain vs. loss frame of the pro-environmental policy message did not affect participants’ agreement with it.

#### Predictors of Agreement With the Message on the Economic Consequences of the Policy

We then proceeded to test whether the gain vs. loss frame of the pro-environmental policy message would affect citizens’ evaluation of the subsequently presented economic impact message (H2) and whether such an effect would be moderated by place identity (H3). We ran a hierarchical linear regression with agreement with the economic impact message as the dependent variable, and three groups of predictors: gender, age, education, political orientation, and risk perception (Step 1); the environmental impact frame (contrast-coded +1 for the gain frame condition and −1 for the loss frame condition), the economic impact frame (contrast-coded +1 for the gain frame condition and −1 for the loss frame condition), and their interaction (Step 2); and place identity and the two- and three-way interactions of place identity with the environmental and economic impact frames (Step 3; Table 5).

**Table 5:** Predictors of participants’ agreement with the statement on the economic impact of the policy (Study 2).

		*β*	*SE*	*β*	*t*	*p*
*Step 1*	(Constant)	6.661	0.570		11.685	0.000
	Gender	−0.180	0.143	−0.057	1.263	0.207
	Age	0.002	0.005	0.020	0.436	0.663
	Education	−0.100	0.025	−0.187	3.968	0.000
	Political orientation	−0.044	0.041	−0.048	1.085	0.278
	Envir. risk perception	−0.146	0.058	−0.112	2.495	0.013
*Step 2*	(Constant)	6.764	0.565		11.971	0.000
	Gender	−0.127	0.142	−0.040	0.893	0.372
	Age	0.002	0.005	0.016	0.351	0.726
	Education	−0.112	0.025	−0.209	4.453	0.000
	Political orientation	−0.036	0.041	−0.039	0.892	0.373
	Envir. risk perception	−0.144	0.058	−0.111	2.506	0.013
	Policy framing	0.057	0.069	0.036	0.821	0.412
	Econ. impact framing	0.191	0.069	0.120	2.751	0.006
	Policy × Econ. impact framing	0.199	0.070	0.125	2.855	0.004
*Step 3*	(Constant)	6.833	0.583		11.716	0.000
	Gender	−0.144	0.143	−0.045	1.008	0.314
	Age	0.002	0.005	0.015	0.333	0.739
	Education	−0.113	0.025	−0.212	4.524	0.000
	Political orientation	−0.039	0.040	−0.042	0.963	0.336
	Envir. risk perception	−0.153	0.068	−0.117	2.252	0.025
	Policy framing	0.050	0.069	0.031	0.725	0.469
	Econ. impact framing	0.181	0.069	0.114	2.640	0.009
	Policy × Econ. impact framing	0.193	0.069	0.121	2.802	0.005
	Place identity	0.051	0.083	0.032	0.617	0.538
	Policy framing × Place identity	0.039	0.069	0.024	0.562	0.575
	Econ. impact framing × Place identity	0.091	0.070	0.057	1.309	0.191
	Policy × Econ. impact framing × Place Id.	0.262	0.069	0.164	3.809	0.000

Policy and economic impact framing coded as +1 gain; −1 loss.

In Step 1, main effects of education, *β* = −0.187, *t* = 3.97, *p* < 0.001, and of environmental risk perception emerged, *β* = −0.112, *t* = 2.50, *p* = 0.013, indicating that the more educated and more environmentally concerned participants were in general less persuaded by any message on the economic impact of the policy. In Step 2, an effect of the economic impact frame, *β* = 0.120, *t* = 2.75, *p* = 0.006, was found, as well as a significant interaction effect with the environmental impact frame, *β* = 0.125, *t* = 2.89, *p* = 0.004. Consistent with our H2, we found that agreement with the message describing the negative economic impact of the policy (loss frame) was significantly higher when the pro-environmental policy was presented with an environmental loss frame than with an environmental gain frame (*M* = 4.71, *SD* = 3.00 and *M* = 3.44, *SD* = 2.41, respectively), *p* < 0.001. Agreement with the message describing the positive economic impact of the policy (gain frame) did not differ between the environmental gain and loss frame conditions (*M* = 8.75, *SD* = 1.96 and *M* = 8.47, *SD* = 2.18, respectively), *p* = 0.410.

**Figure 2:**
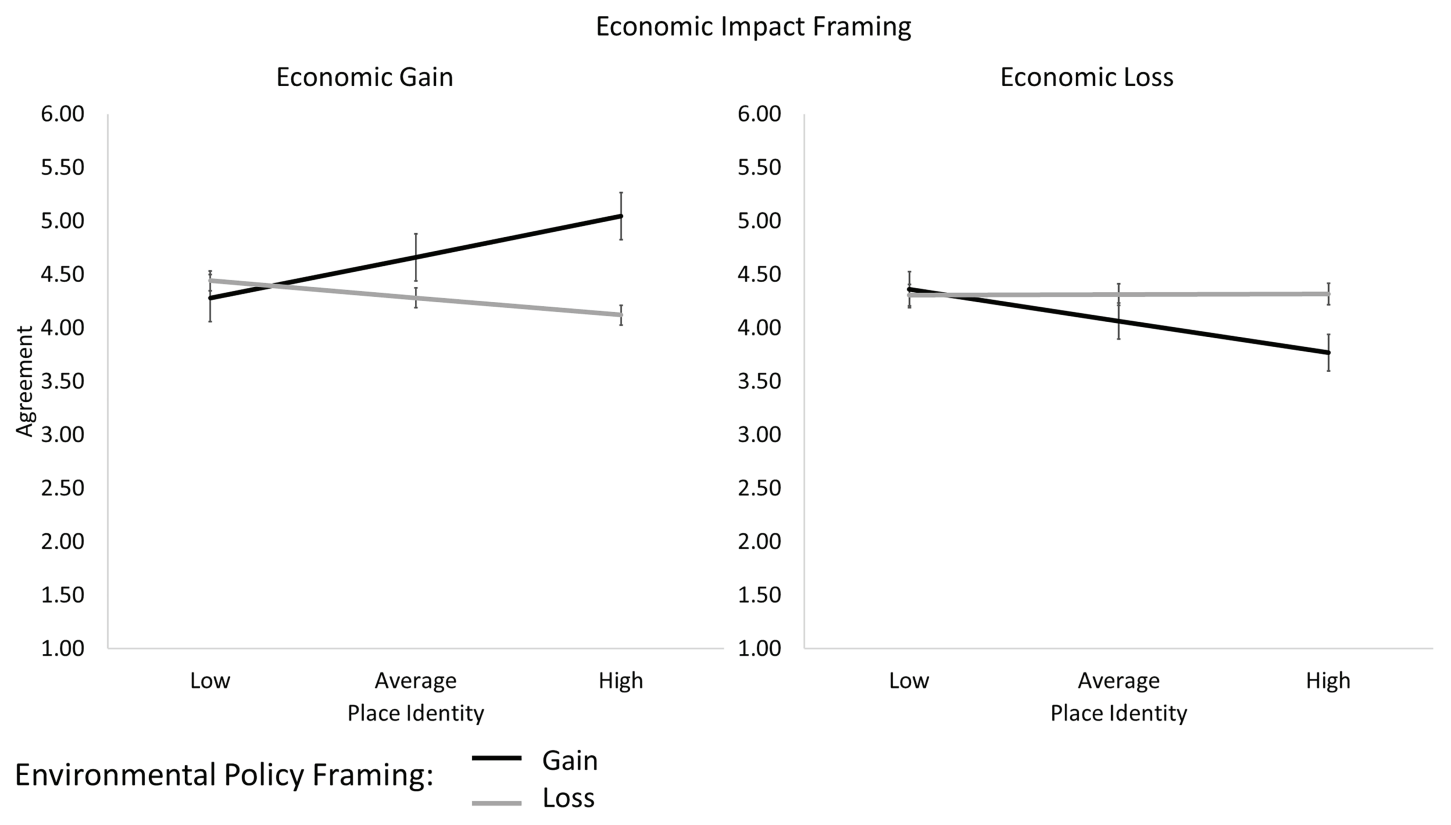
Agreement with gain vs. loss messages on the economic impact of the policy as a function of gain vs. loss framing of the environmental policy and place identity (Study 2).

Finally, in Step 3, a significant three-way interaction effect was found, *β* = 0.164, *t* = 3.81, *p* < 0.001. We probed this interaction with follow-up conditional effects analyses (setting the place identity score at the conventional −1 *SD*, *M*, and +1 *SD* values), and found that the interaction between the environmental and economic frames was significant among participants with average, *β* = 0.19, *t* = 2.74, *p* = 006, or strong place identity, *β* = 0.44, *t* = 4.39, *p* < 0.001, but not among participants with weak place identity, *β* = −0.05, *t* = 0.53, *p* = 0.600 (Figure 2). Conditional testing of the two manipulated variables also showed no effects of either environmental or economic impact frames at low levels of place identity, *β*s < 0.09, *t*s < 0.58, *p*s > 0.566. These results confirmed our H3, according to which the moderating effect of the environmental loss frame on the persuasiveness of the economic loss frame would be further increased among participants with strong place identity. In addition to the hypothesized interaction effect on the agreement with the loss-framed message on the economic impact of the policy, we also observed an opposite interaction effect on the agreement with the gain-framed message on the economic impact of the policy. The agreement with the potential economic benefits deriving from the policy was higher among strongly identified participants who had read the gain-framed description of the environmental consequences of the policy, suggesting that this group of participants was particularly sensitive to related communication in both directions.

To sum up, the results of Study 2 complemented those of Study 1, showing that whereas support for the message on the pro-environmental policy was mainly driven by individual characteristics of the audience, such as risk perception and place identity, support for the message on its economic impact was influenced by subtle but relevant communicative factors. When both the environmental and the economic impact messages were framed in terms of losses, they were met with significantly higher levels of agreement, and this effect was even more evident among participants who were highly identified with their place.

## General Discussion

In two studies, we investigated the factors influencing how citizens evaluate messages highlighting the economic impact of a pro-environmental policy. The policy we examined concerned the introduction of a natural reserve to reduce excessive land use in a lakeside area. Our hypotheses were tested first with a large sample of Italian citizens (Study 1) and then with a sample of inhabitants of a lake area in Northern Italy (Study 2). Results supported our expectation that citizens’ agreement with messages that highlight the *negative* economic effects of pro-environmental policies may be increased by two communicative strategies that (paradoxically) are instead often used to *promote* this kind of policy, i.e., referring to the local dimension of the policy and framing the policy as a way to avoid serious negative environmental consequences.

These results advance our knowledge of the factors that influence the effectiveness of communication on pro-environmental policies and offer indications on how to improve such communication.

In both our studies, participants were generally very favourable toward the proposed pro-environmental policy, and such attitude was strongly associated with environmental risk perception. This finding corroborates previous research indicating that the public awareness of environmental problems, such as pollution, climate change, and excessive land use, is the key factor needed to gain initial support for this type of policies (Lorenzoni and Pidgeon, 2006; Lee et al., 2015). This is particularly relevant with environmental issues that are not immediately apparent to most citizens, either because of their slow and progressive onset (as in the case of climate change) or because they are intertwined with other complex issues, such as urban, agricultural, and industrial planning (as in the case of land use considered in this research).

Overall, our results show that the way the pro-environmental policy and its impact are framed does make the difference. Even when agreement with the proposed policy is generally high, as was the case in our two studies, under given conditions messages stressing the negative economic impact of the policy can be very persuasive, and potentially undermine support for said policy. The main contribution of our research is precisely having highlighted some of these conditions, and a relevant individual difference factor (place identity) moderating this effect.

First, our results show that adding reference to the local dimension in messages focused on the negative economic impact of a pro-environmental policy enhances agreement with these messages (Study 1). Previous research has investigated reference to the local dimension and impact of pro-environmental issues as a communicative strategy to *promote* citizens’ awareness of environmental problems, and support for pro-environmental policies (Brügger et al., 2015; Messling et al., 2015). We found that this communicative strategy may potentially backfire when the economic side of the policy impact is put under scrutiny, as it is often the case. This could reflect the increased scrutiny prompted by the possibility of negative repercussions for one’s own area. It may also depend on an objective assessment of the quality of the argument (as economic repercussions of a land use policy realistically impact only a limited area). In any case, the proponents of pro-environmental policies should be aware that whereas a stress on the local relevance of the policies can be beneficial, a stress on the local economic impact of the same policies can instead rise citizens’ concern for the financial welfare of their local community.

Second, our results show that communication presenting the effects of a pro-environmental policy in terms of avoidance of future environmental damage, rather than in terms of future environmental gain, makes recipients more sensitive to the risk of economic damage as well, thus potentially hindering overall support for the proposed policy (Study 2). Similar interactive framing effects has been observed in previous research on climate change policies (Bertolotti and Catellani, 2014; Moser, 2016), but in our research for the first time they have been found to affect evaluations across different domains, i.e., environmental frames affecting economic evaluation, and vice-versa. Therefore, messages focusing on how the adoption of a given policy can prevent negative environmental consequences may have some unintended effects, such as making audiences somewhat sensitive to other types of risks as well, such as economic and financial risks.

Third, we found that recipients’ place identity can moderate the effects of communication about pro-environmental policies. In our research, participants with strong place identity were especially sensitive to matching frames regarding the environmental and the economic consequences of the policy (Study 2). This result complements what was shown in Study 1 regarding the effect of reference to the local dimension within the message on the economic impact. As past research has suggested, strong local identity and place attachment can sometimes result in opposition to pro-environmental plans (Devine-Wright, 2009; Devine-Wright and Howes, 2010). Our findings indicate that economic concerns play a role in this potential backlash, as citizens are understandably interested not only in preserving the environment in which they live, but also in the economic welfare of the local community. Nevertheless, we also found that communication stressing both the environmental and economic advantages of pro-environmental policies was particularly persuasive for individuals with a strong place identity. Anyone who proposes a pro-environmental policy emphasising its relevance at the local level must therefore be careful to stress both the environmental *and* economic gains expected from this policy, if they want to avoid that reference to the local dimension ends up having an opposite effect to the desired one.

Our research has some limitations. One potential issue is the limited space allocated to the messages used in our survey experiments. This may have limited the ecological validity of our findings, since actual public debates on pro-environmental policies usually provide citizens with more arguments, allowing them to make more nuanced and thoughtful evaluations than the ones they quickly expressed in our studies. At the same time, it is often the case that citizens in real life read short statements or watch quick soundbites discussing the expected effects of a policy. From this point of view, our messages can be considered indeed as rather realistic. Another potential limitation is related to the fact that in both our studies participants were asked to express their opinions on the policy *before* any information on the economic impact was mentioned. Future research may reverse the order of presentation of the messages, to investigate whether messages on the economic impact of the policy (and their framing) can similarly affect citizens’ agreement with messages on its environmental impact. Both our studies had a sizable number of participants, with Study 1 involving a large nation-wide sample of citizens and Study 2 involving citizens of a single geographic area. This allowed us to test the effects of communication on a pro-environmental policy proposal on actual citizens who may have dealt (or may deal in the future) with similar proposals. Further research in different areas and different nations is however needed to explore the role of geographic, environmental, social, and economic contingencies in the process we have analysed.

To conclude, our research provides new insights on how to communicate about the costs and benefits of pro-environmental policies. Messages promoting the adoption of such policies could effectively anticipate certain common and compelling counterarguments by avoiding frames that make citizens too sensitive to negative economic concerns.

## Data Availability Statement

The raw data supporting the conclusions of this article will be made available by the authors, without undue reservation.

## Ethics Statement

The studies involving human participants were reviewed and approved by Psychology Research Ethics Committee (CERPS); Department of Psychology, Catholic University of Milan. Written informed consent for participation was not required for this study in accordance with the national legislation and the institutional requirements.

## Author Contributions

MB: conceptualization and writing of the manuscript. PC: supervision, conceptualization, and writing. Both the authors contributed to the article and approved the submitted version.

### Conflict of Interest

The authors declare that the research was conducted in the absence of any commercial or financial relationships that could be construed as a potential conflict of interest.

## Footnotes

^1^The ITANES group has been analyzing the Italians’ voting behavior since the beginning of 1990s. Readers interested in this research program should visit the website www.itanes.org. The 2018 ITANES survey was conducted prior to the March 4th Italian political election and involved a representative sample of Italian voters.

^2^The CLIC-PLAN project (CLImate Change. Local adaptation PLAN for lake municipalities in the subalpine territory with a strong tourist vocation) was a multidisciplinary research project conducted between 2017 and 2019 by the Catholic University of Milan. The main aims of the project were to investigate attitudes toward climate change among citizens of a tourism-oriented lakeside area, and to develop and communicate a local climate adaptation plan.

^3^The questionnaire included also some additional measures of participants’ beliefs in climate change, and of pro-environmental individual and collective action intention. All three measures were positively and strongly correlated with both the agreement with the pro-environmental policy message, *r*s(498) > 0.450, *p*s < 0.001, and with environmental risk perception, *r*s(498) > 0.525, *p*s < 0.001. To reduce the complexity of the model and keep the number of predictors low, these variables were omitted from the regression models presented here.
